# Supplementary material for: Enhancing photosynthetic CO2 fixation by assembling metal-organic frameworks on Chlorella pyrenoidosa
Source: Nat Commun. 2023 Sep 2;14:5337. doi: 10.1038/s41467-023-40839-0 (PMC10475011; doi:10.1038/s41467-023-40839-0)
Supplement: Supplementary file 1 — Supplementary Information [file 41467_2023_40839_MOESM1_ESM.pdf]

Enhancing photosynthetic CO_2 fixation by assembling metal-organic frameworks on *Chlorella pyrenoidosa*

Li *et al.*

Supplementary Method 1. Additional characterizations of MOF and algae

XRD patterns of NH_2-MIL-101-Fe

XRD patterns of NH_2-MIL-101-Fe were plotted by D/MAX 2500/PC X-ray Powder diffractometer (Rigaku, Japan) using Cu Kα radiation (40 kV, 100 mA). Scan range: 0.5° ~ 50°, scan rate: 1°/min.

The N_2 ad/desorption isotherms of NH_2-MIL-101-Fe

The N_2 ad/desorption isotherms of NH_2-MIL-101-Fe were plotted by ASAP2020 physical adsorption analyzer (Micromeritics, USA) to determine the surface area and pore volume of MOF. 100 mg MOF powder in tube was degassed 2 h under high vacuum and 100℃, and then measured its N_2 adsorption isotherms at 77 K.

Size and zeta potential measurements

NH_2-MIL-101-Fe and *C. pyrenoidosa* were suspended and diluted by water to appropriate concentrations. After pH adjustments (3 ~ 12), their zeta potentials were measured using Zetasizer Nano ZS (Malvern, UK). Their hydration diameters were measured on the same instrument with size mode.

Photosynthetic oxygen evolution measurements

A Clark-type oxygen electrode (Strathkelvin SI130, UK) was used to measure the photosynthetic oxygen evolution activity of *C. pyrenoidosa* and MOF/*C. pyrenoidosa*. Cell suspensions (3 mL, OD_{750} = 0.6) was added to the oxygen electrode chamber with 2 mM HCO_3^-. The mixture was purged with nitrogen for 5 min to remove the dissolved oxygen, then the real-time oxygen concentration in the chamber was monitored under the irradiation of 50 or 1000 μE

m^{-2} s^{-1} red light (λ > 600 nm, 24℃). The average oxygen evolution rate between 3 min and 7 min during the kinetic plots were calculated as the oxygen evolution activity.

For the measurements of the apparent affinity of the photosynthetic O_2 production for C_i (CO_2 or HCO_3^-) of *C. pyrenoidosa* and MOF/*C. pyrenoidosa* cells, the above method was modified, the microalgal cells were centrifuged for concentration, and redispersed in the supernatants of media (after O_2 depletion by Ar purging) to simulate their cultivation condition. For different HCO_3^- concentrations, 1 M HCO_3^- stock solution was diluted to different concentrations in chamber. For different CO_2 concentrations, a serias of diluted solution of CO_2-saturated water (1450 ppm DIC) were regarded as the solutions of different CO_2 concentrations. The average O_2 evolution rate between 40 s and 100 s during the kinetic plots were recorded as the net O_2 evolution rate.

Flow cytometry

MOF, *C. pyrenoidosa* and MOF/*C. pyrenoidosa* were redispersed in BG-11 medium to be analyzed by a Sony SH800 flow cytometer. The cell density of *C. pyrenoidosa* was 0.2×10^7 cell ml^{-1}, and the concentration of MOFs is 10 ppm. The sheath fluid is 10 mM phosphate buffer solution (pH 7.2). FSC (forward scatter) mode was used for MOF and *C. pyrenoidosa* to analyze the particle size distribution of those sample suspensions according to their FSC-area signals (analyzed in Cell Sorter Software version 2.1).

UV-Vis absorption characterization

MOF (50 ppm), *C. pyrenoidosa* (OD_{750} = 0.32) and *C. pyrenoidosa/*MOF were redispersed in BG-11 medium. Their absorbances (350 ~ 800 nm) were recorded by a V-650 UV-Vis spectrophotometer (JASCO, Japan). For MOF/*C. pyrenoidosa* sample, after 30 min co-cultivation

of MOF and *C. pyrenoidosa*, the suspension was centrifuged (2220 g, 5 min) to remove excess MOF particles before UV-Vis absorption characterization.

Chlorophyll content determination

C. pyrenoidosa and MOF/*C. pyrenoidosa* cell suspensions cultivated for 2 days were centrifugated and rinsed twice, then diluted to a final OD_{750} of 1.0 by deionized water. 1 mL cell suspension was centrifugated and replaced the solvent to anhydrase ethanol, the extracted the pigments for 12 hours, and record the UV-Vis absorption spectra (400 ~ 800 nm) of the supernatants. The contents of chlorophyll and carotenoid were calculated as:

$$Chl\ a\ (\mu g\ mL^{-1}) = 13.95 \times A_{665} - 6.88 \times A_{649} \quad (1)$$

$$Chl\ b\ (\mu g\ mL^{-1}) = 24.96 \times A_{649} - 7.32 \times A_{665} \quad (2)$$

$$Carotenoid\ (\mu g\ mL^{-1}) = \frac{1000 \times A_{470} - 3.27 \times Chl\ a - 104 \times Chl\ b}{229} \quad (3)$$

Chl a, Chl b and Cx are chlorophyll a, chlorophyll b and carotenoid, respectively.

Measurement of dissolved inorganic carbon

The adsorption of dissolved inorganic carbon (DIC) in MOF was determined by a TOC/TN Analyzer (Teledyne Tekmar, USA). Phosphoric acid was used to convert bicarbonate/carbonate to CO_2, then release from the solution and be measured with an IR detector. In BG-11 medium, there was negligible organic and inorganic carbon source in fresh BG-11 medium before aeration. Ambient air was bubbled in BG-11 for 30 min, then a series amount of MOF suspensions was dispersed in it. After 30 min adsorption, the supernatants were obtained by centrifugation (12000 g, 5 min), then be diluted 5 times for inorganic carbon measurement. A series of sodium bicarbonate solutions were tested in advance and a calibration curve of DIC was mapped.

Measurement of ligand, CA inhibitors in medium

The ligand of NH_2-MOF (NH_2-BDC), two kinds of CA inhibitors (AZA and EZA) in medium were analyzed by high performance liquid chromatograph (HPLC) (Agilent 1200, USA) equipped with ultraviolet detector using Unitary C18 column (5 μm, 4.6 ×150 mm) (Agilent Ltd.) at 35 °C. The flow rate of the mobile phase was set as 0.8 mL/min. The injection volume of HPLC was 10 μL. The detection wavelength of UV detector was 352 nm and the mobile phase was acetonitrile and 0.1% H_3PO_4 aqueous solution (70:30, %, v/v).

Measurement of CA activity

The enzymatic activities of extracellular carbonic anhydrase (CA) existed in the supernatant of microalgal suspensions were determined with a colorimetric CA activity assay kit (Solarbio, China). Briefly speaking, 0.7 mL of Tris buffer (50 mM, pH 7.5), 0.2 mL of p-nitrophenyl acetate (p-NPA) aqueous solution, and 0.1 mL of medium centrifuged from different microalgal suspensions (or the redispersed suspension of MOF pretreated in medium) were mixed in the quartz cuvette. Since the hydrolysis of p-NPA to p-nitrophenol (p-NP) can be catalyzed by CA, the increase rate in the concentration of p- (p-NP) indicates the enzymatic activity of CA. The absorbances at 405 nm of the mixture were recorded before and after 5 minutes' incubation in water bath (37 °C) to calculate the CA activity. The self-dissociation of p-NPA was measured in the same conditions for correction. The concentration of p-NP was calibrated by p-NP standard solutions.

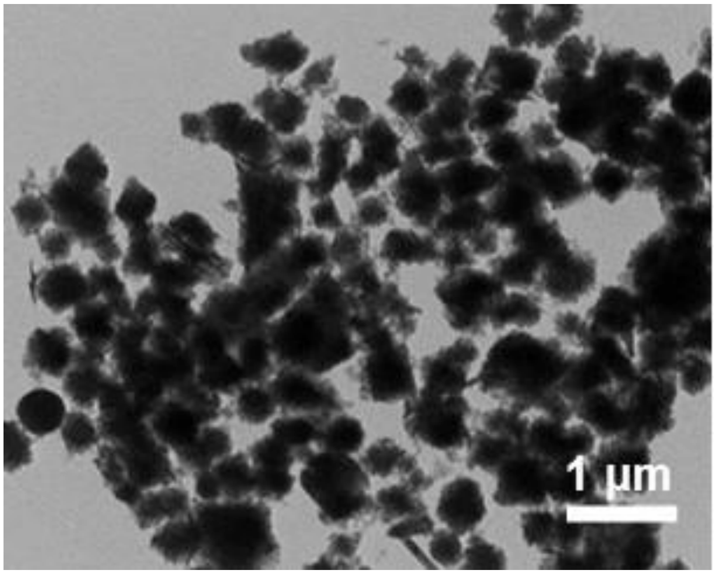

Supplementary Figure 1. TEM image of MOF NH_2-MIL-101-Fe.

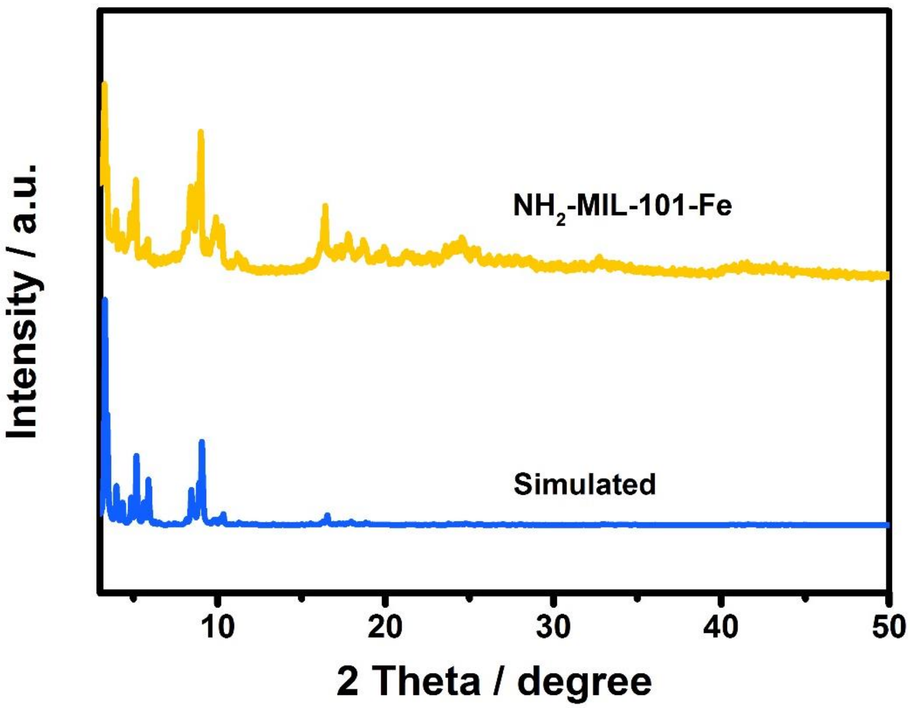

Supplementary Figure 2. Experimental and simulated XRD pattern of MOF NH_2-MIL-101-Fe. Source data are provided as a Source Data file.

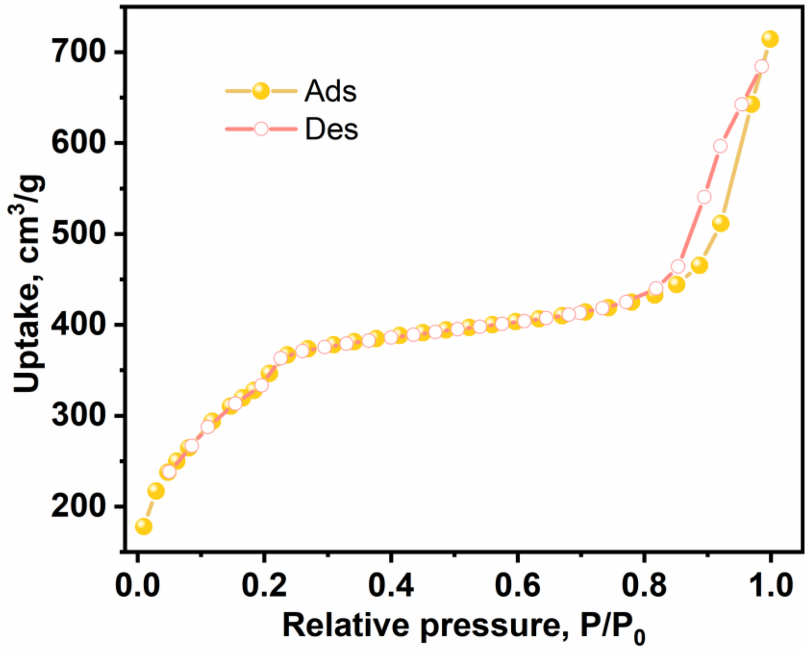

Supplementary Figure 3. The N_2 ad/desorption isotherms of NH_2-MIL-101-Fe at 77 K. Source data are provided as a Source Data file.

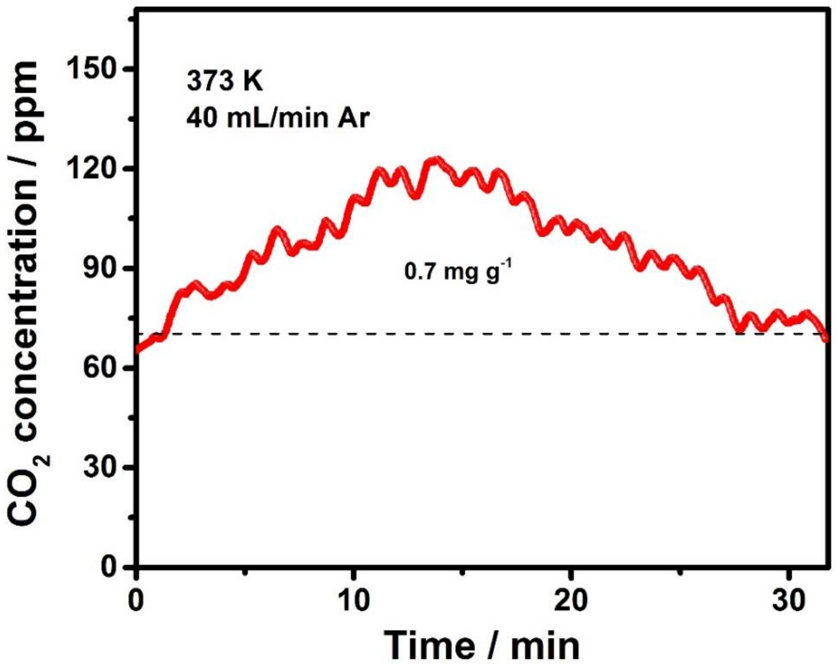

Supplementary Figure 4. The CO_2 desorption curve of MOF at 373 K. MOF in water adsorbed CO_2 from purged air, the CO_2 uptake amount was determined by thermal desorption. (Temperature, 373 K; Flux, 40 ml/min Ar; MOF amount, 50 mg). Source data are provided as a Source Data file.

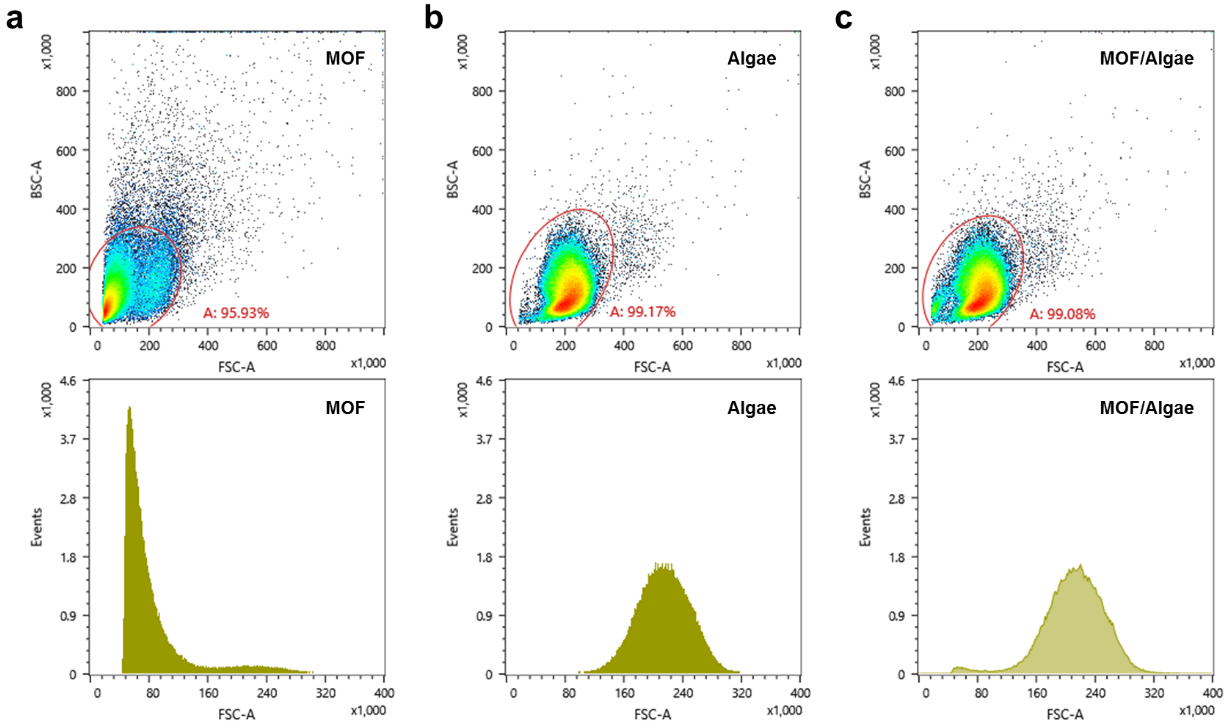

Supplementary Figure 5. Flow cytometric scatter plot and FSC-A distribution. (a) MOFs, (b) *C. pyrenoidosa* and (c) *C. pyrenoidosa* in the presence of MOFs. Cell density of *C. pyrenoidosa*, 0.2×10^7 cell ml^{-1}. Source data are provided as a Source Data file.

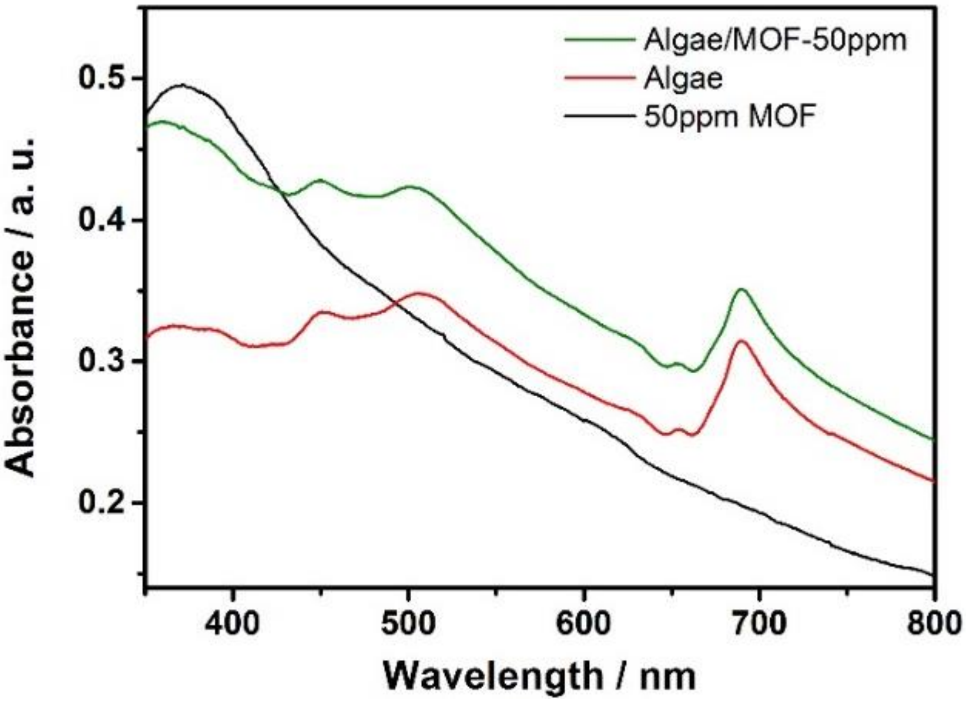

Supplementary Figure 6. UV-Vis absorption spectra of MOF and *C. pyrenoidosa* with or without MOF. OD_{750} of *C. pyrenoidosa*, 0.32; MOF amount, 50 ppm. Source data are provided as a Source Data file.

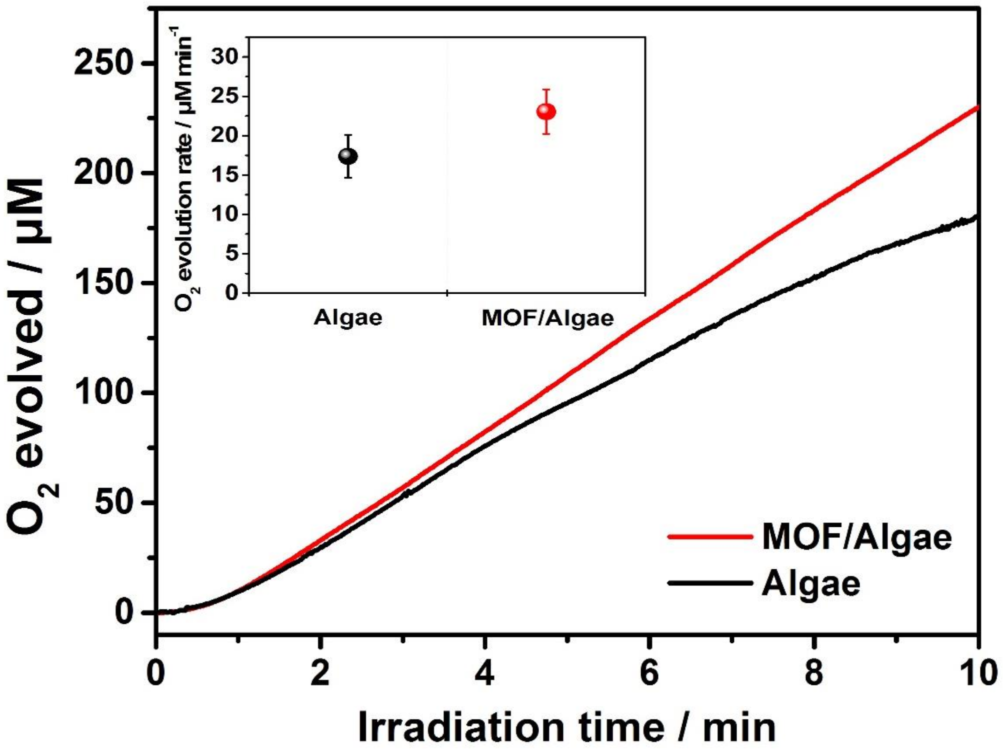

Supplementary Figure 7. Photosynthetic oxygen evolution of *C. pyrenoidosa* in the absence and in the presence of MOF. Light intensity, 1000 μE m^{-2} s^{-1} (λ > 600 nm). Error bars represent the standard deviation of the results from three biologically independent experiments. Source data are provided as a Source Data file.

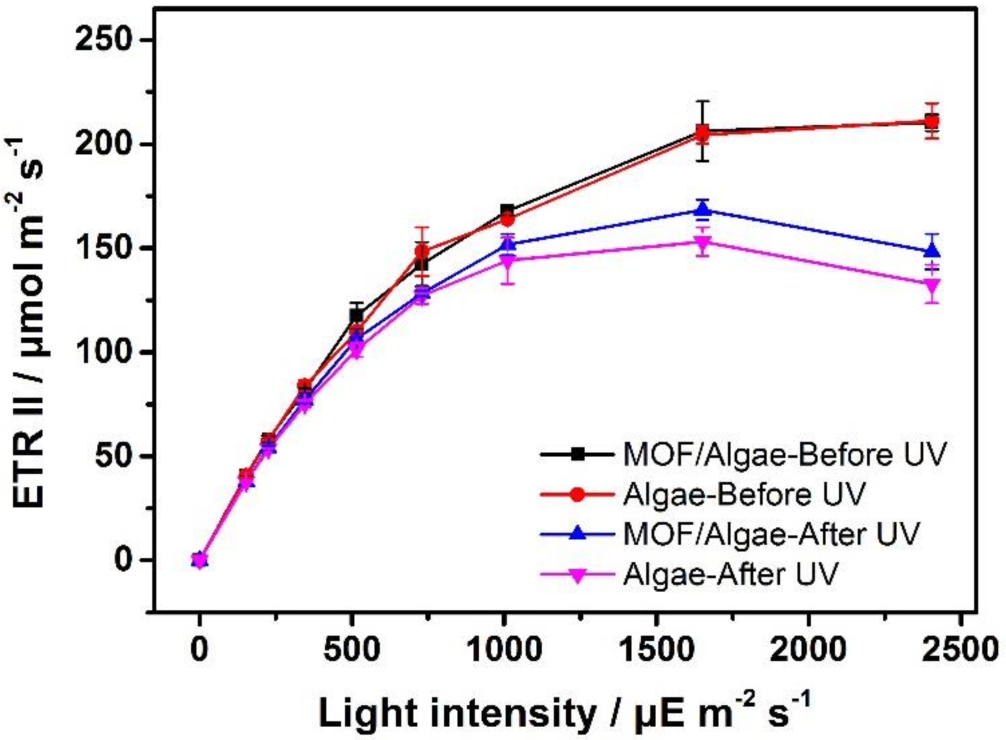

Supplementary Figure 8. The plots of electron transfer rates of PSII versus light intensities of *C. pyrenoidosa*. In the absence and in the presence of MOF before and after 30 min 10 μE m^{-2} s^{-1} UV irradiation (λ = 254 nm). Error bars represent the standard deviation of the results from three biologically independent experiments. Source data are provided as a Source Data file.

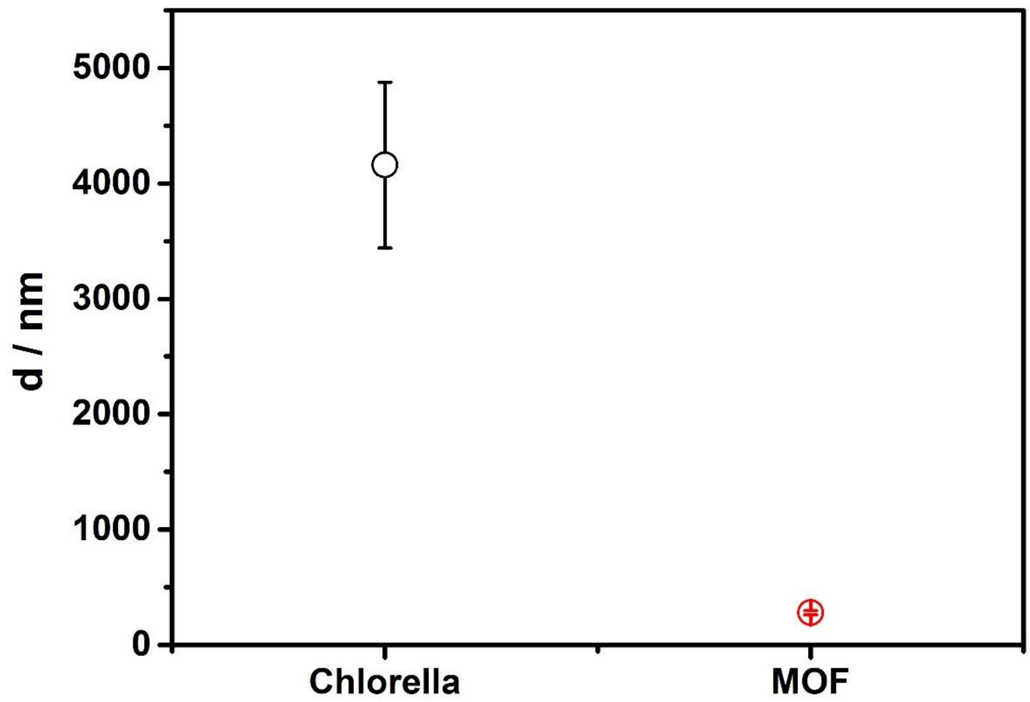

Supplementary Figure 9. Size measurements of *C. pyrenoidosa* and NH_2-MOF. Dynamic light scattering method was applied. OD_{750} of *C. pyrenoidosa*, 0.20; MOF amount, 10 ppm. Error bars represent the standard deviation of the results from three biologically independent experiments. Source data are provided as a Source Data file.

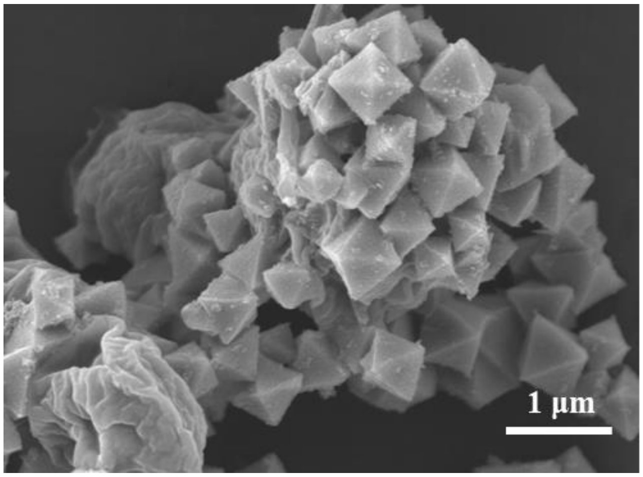

Supplementary Figure 10. SEM image of *MOF/C. pyrenoidosa* after two-day cultivation. Cultivation conditions: temperature, 26°C; light intensity, 50 µE m^{-2} s^{-1}; 20 mL min^{-1} ambient air flow (LC); 24 hours continuous illumination.

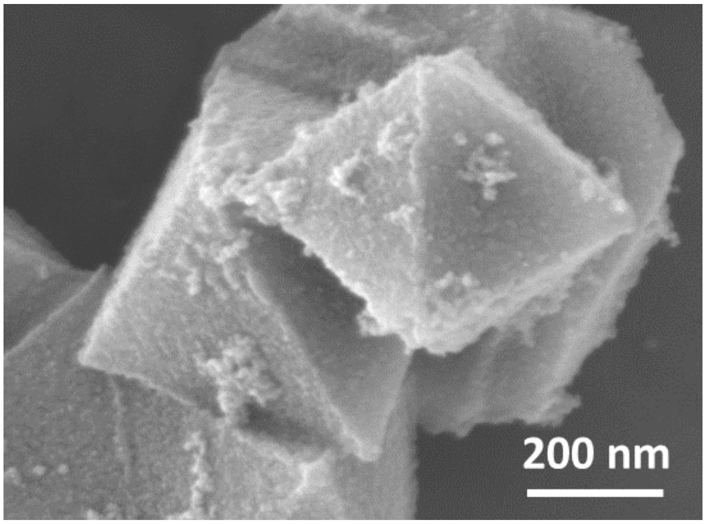

Supplementary Figure 11. SEM image of MOF pretreated in the supernatant of *C. pyrenoidosa* suspension after two-day cultivation. OD_{750} of *C. pyrenoidosa*, 1.5; MOF amount, 1.5 mg.

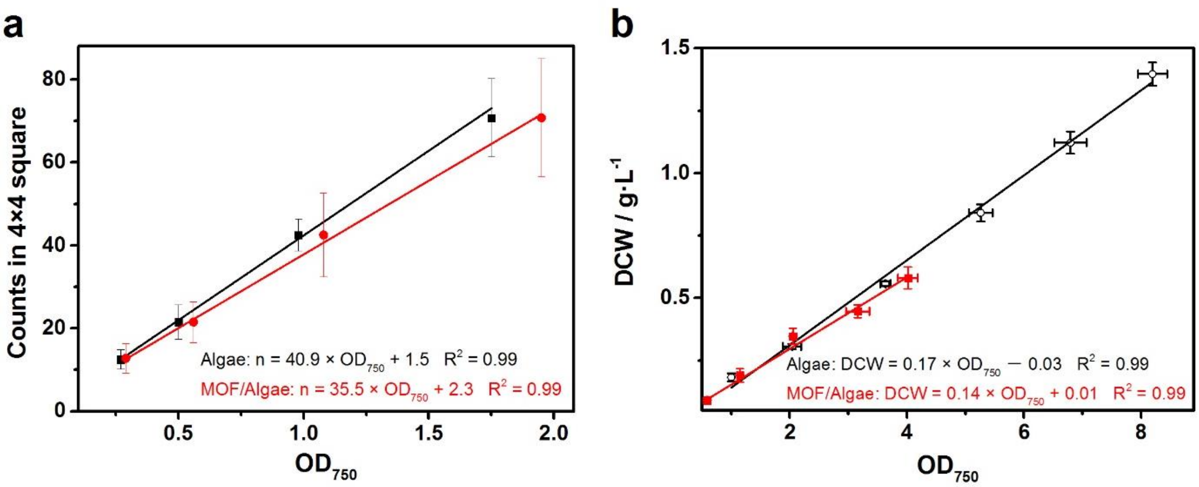

Supplementary Figure 12. The calibration curves of cell density and biomass versus OD_{750}. **(a)** algal cell number (on 0.2×0.2 μm square) versus the optical density at 750 nm (OD_{750}), and **(b)** the dry cell weight (DCW, net biomass without water) of *C. pyrenoidosa* in the absence and in the presence of MOF. Error bars in **(a-b)** represent the standard deviation of the results from three biologically independent experiments. Source data are provided as a Source Data file.

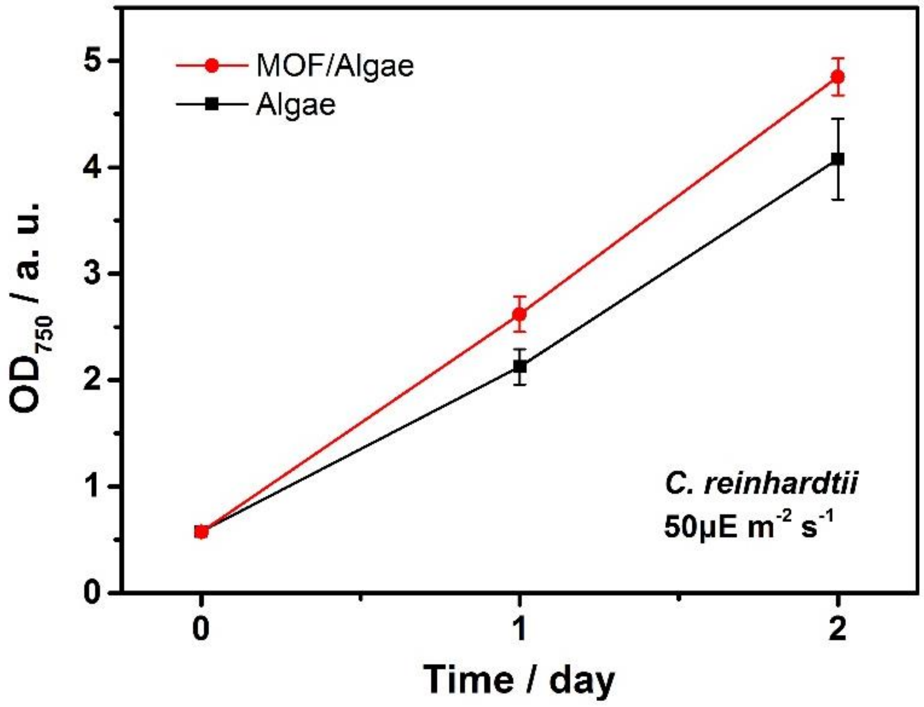

Supplementary Figure 13. The growth curves of *Chlamydomonas reinhardtii*. With the addition of 50 ppm MOF at initial pH 7 and be aerated with ambient air. Cultivation conditions: temperature, 26°C; light intensity, 50 μE m^{-2} s^{-1}; 20 mL min^{-1} ambient air flow; 24 hours continuous illumination. Error bars represent the standard deviation of the results from three biologically independent experiments. Source data are provided as a Source Data file.

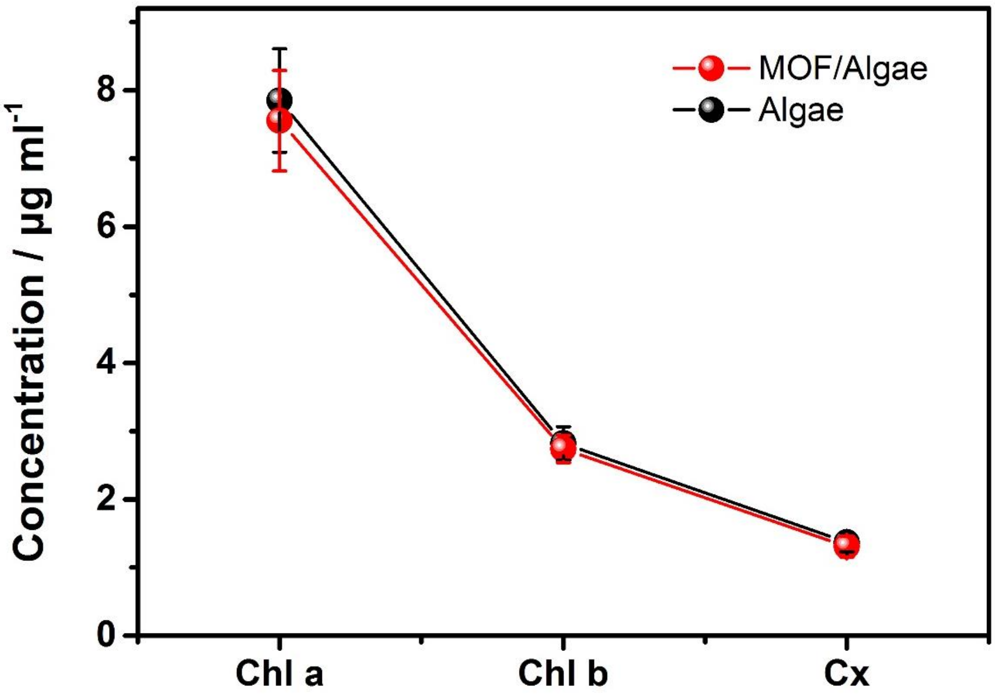

Supplementary Figure 14. The contents of photosynthetic pigments of *C. pyrenoidosa*. Cells were cultivated in the absence and in the presence of MOF after two-day cultivation and extracted by anhydrous ethanol. Chl a, chlorophyll a; Chl b, chlorophyll b; Cx, carotenoids. Error bars represent the standard deviation of the results from three biologically independent experiments. Source data are provided as a Source Data file.

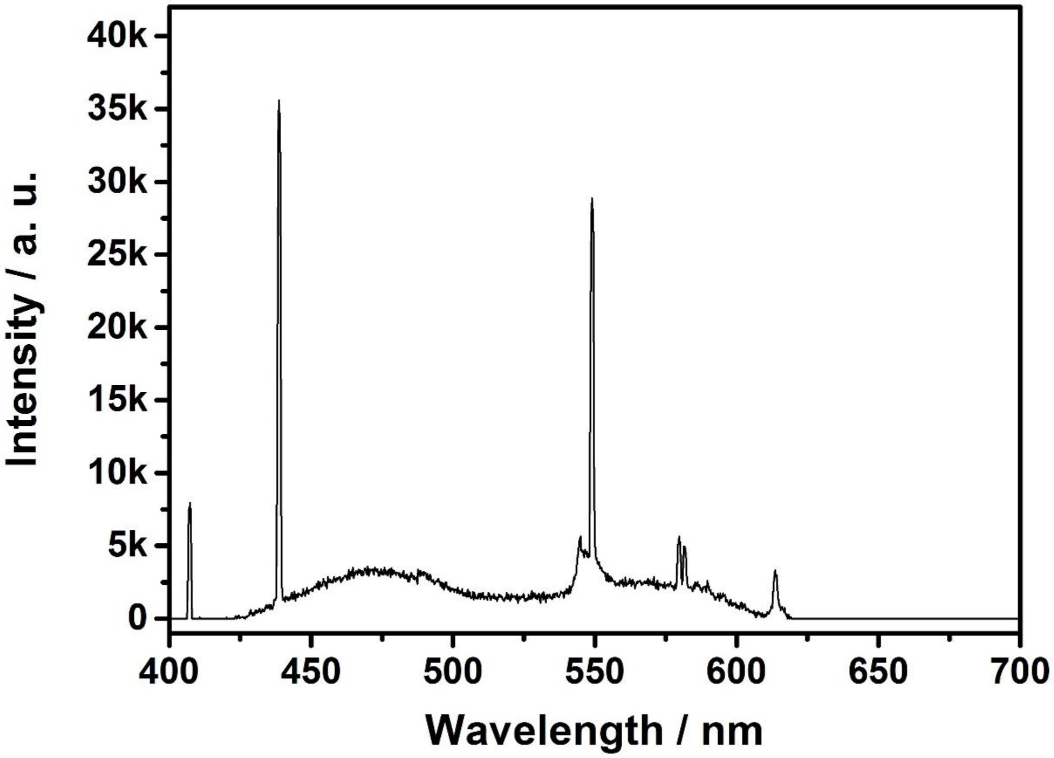

Supplementary Figure 15. The light spectrum of the light source for the cultivation of *C. pyrenoidosa*. Source data are provided as a Source Data file.

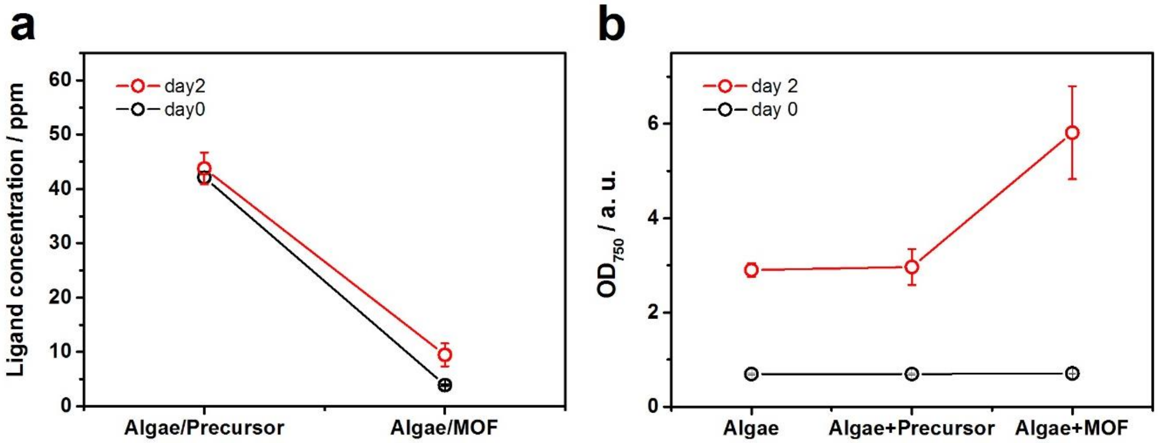

Supplementary Figure 16. Stability of MOF on *C. pyrenoidosa*. (a) The ligand concentration in the media and (**b**) the biomass growth of *C. pyrenoidosa*, *C. pyrenoidosa* with 50 ppm MOF or the same molar MOF precursors, NH_2-BDC + Fe(III) (the organic ligand of MOF and ferric ammonium citrate, respectively) before and after two-day cultivation. Cultivation conditions: temperature, 26°C; light intensity, 50 μE m^{-2} s^{-1}; 20 mL min^{-1} ambient air flow (LC); 24 hours continuous illumination. Error bars in **(a-b)** represent the standard deviation of the results from three biologically independent experiments. Source data are provided as a Source Data file.

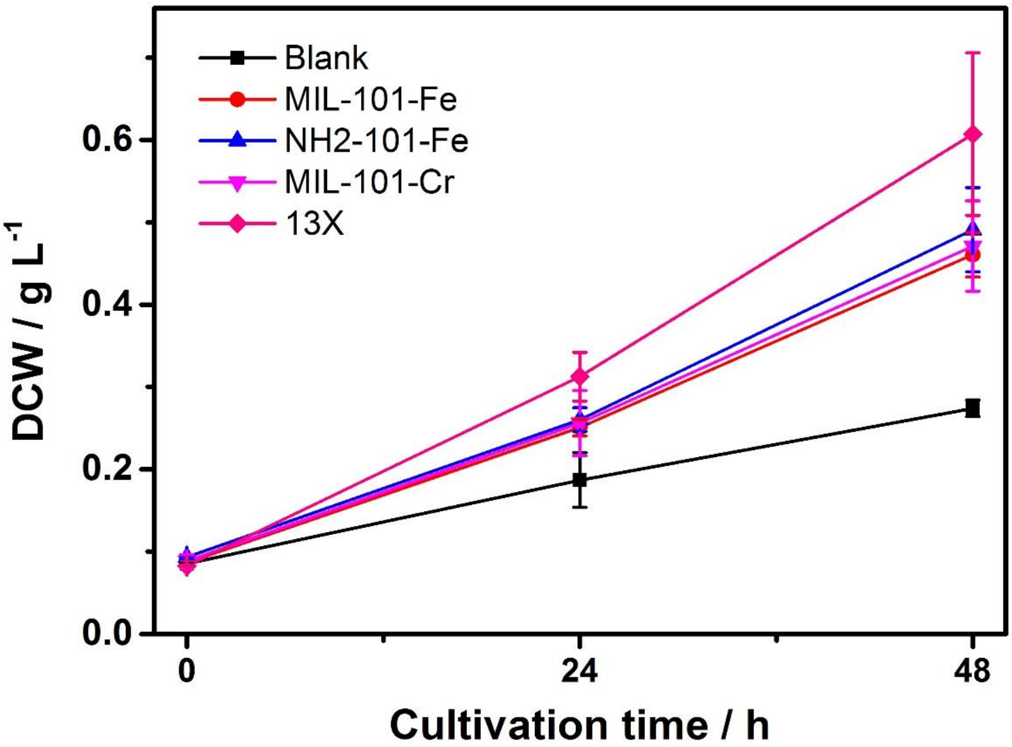

Supplementary Figure 17. Biomass growth of bare *C. pyrenoidosa* and *C. pyrenoidosa* with three kinds of MOFs. MIL-101-Fe, NH_2-MIL-101-Fe and MIL-101-Cr, and one kind of commercial CO_2-adsorption zeolite 13X. Cultivation conditions: temperature, 26°C; light intensity, 50 μE m^{-2} s^{-1}; 20 mL min^{-1} ambient air flow (LC); 24 hours continuous illumination. Error bars represent the standard deviation of the results from three biologically independent experiments. Source data are provided as a Source Data file.

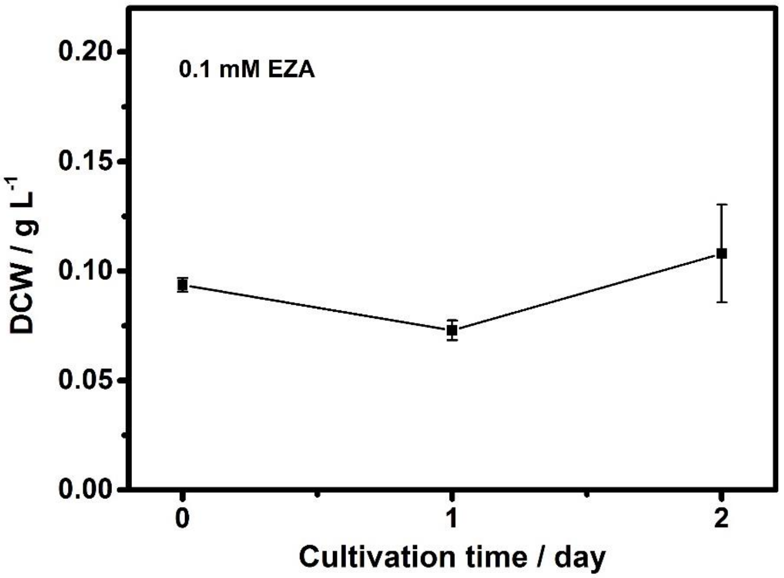

Supplementary Figure 18. Biomass growth curve of *C. pyrenoidosa* during two-day cultivation. 0.1 mM EZA (ethoxazolamide, an internal CA inhibitor) was added at pH 7 under air. Cultivation conditions: temperature, 26°C; light intensity, 50 μE m^{-2} s^{-1}, gas flux, 20 mL min^{-1}; 24 hours continuous illumination. Error bars represent the standard deviation of the results from three biologically independent experiments. Source data are provided as a Source Data file.

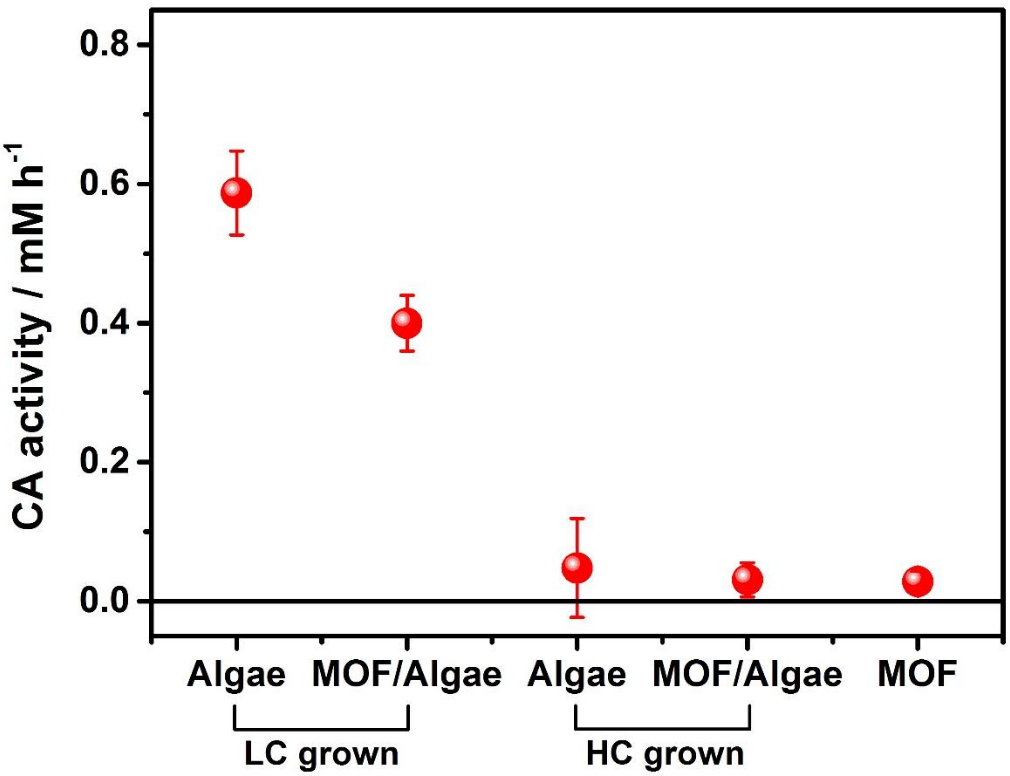

Supplementary Figure 19. CA activities of media or MOF. *C. pyrenoidosa* and MOF/*C. pyrenoidosa* cells were grown under air (LC, 0.04%) and 2% CO_2 (HC). Cultivation conditions: temperature, 26°C; light intensity, 50 μE m^{-2} s^{-1}; 20 mL min^{-1} gas flow; 24 hours continuous illumination. Error bars represent the standard deviation of the results from three biologically independent experiments. Source data are provided as a Source Data file.

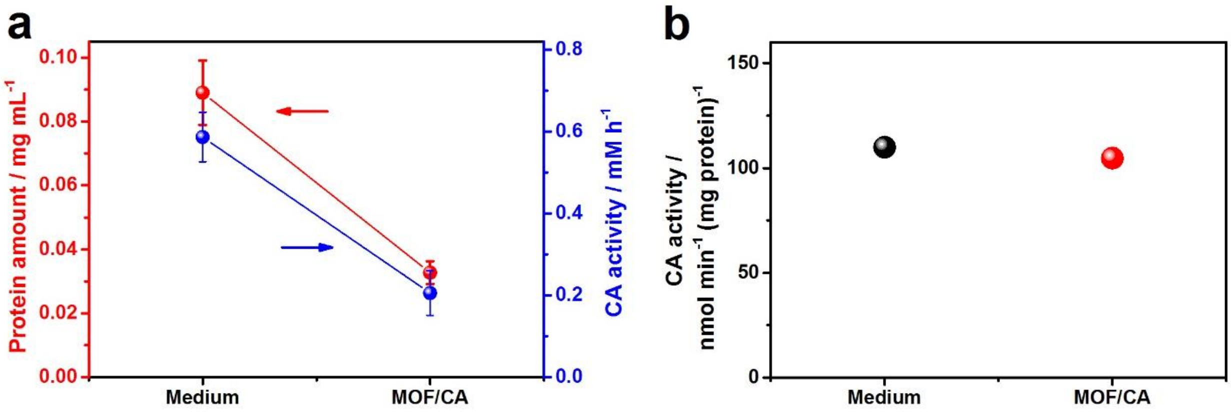

Supplementary Figure 20. The adsorption of extracellular carbonic anhydrase on MOF. (a) Protein amount and the activity of CA in the medium of microalgal suspension (Medium) and adsorbed by MOF particles (MOF/CA). **(b)** Relative activity of CA in the medium of *C. pyrenoidosa* suspension (Medium) and the MOF particles that adsorbed CA in the supernatant of microalgal suspension (MOF/CA). OD_{750} = 1.5, m(MOF) = 1.5 mg. Error bars in **(a-b)** represent the standard deviation of the results from three biologically independent experiments. Source data are provided as a Source Data file.

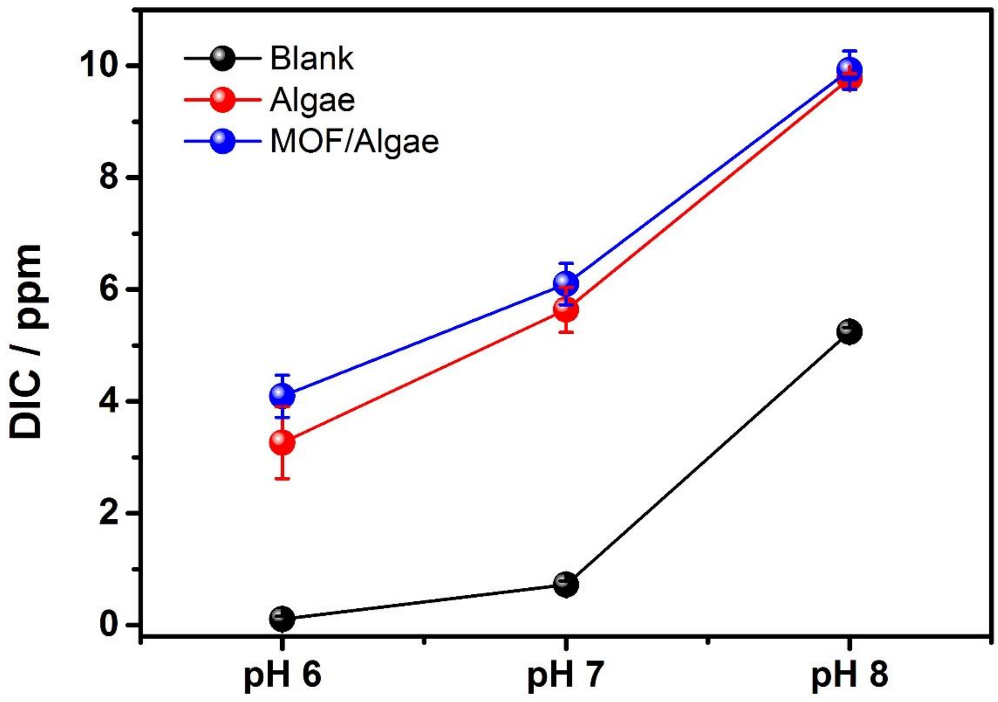

Supplementary Figure 21. The contents of dissolved inorganic carbon in medium. Cells were cultivated in the absence and presence of MOF under different initial pH value before (Blank, only medium without algal cells) and after two-day cultivation (Algae and MOF/Algae). Error bars represent the standard deviation of the results from three biologically independent experiments. Source data are provided as a Source Data file.

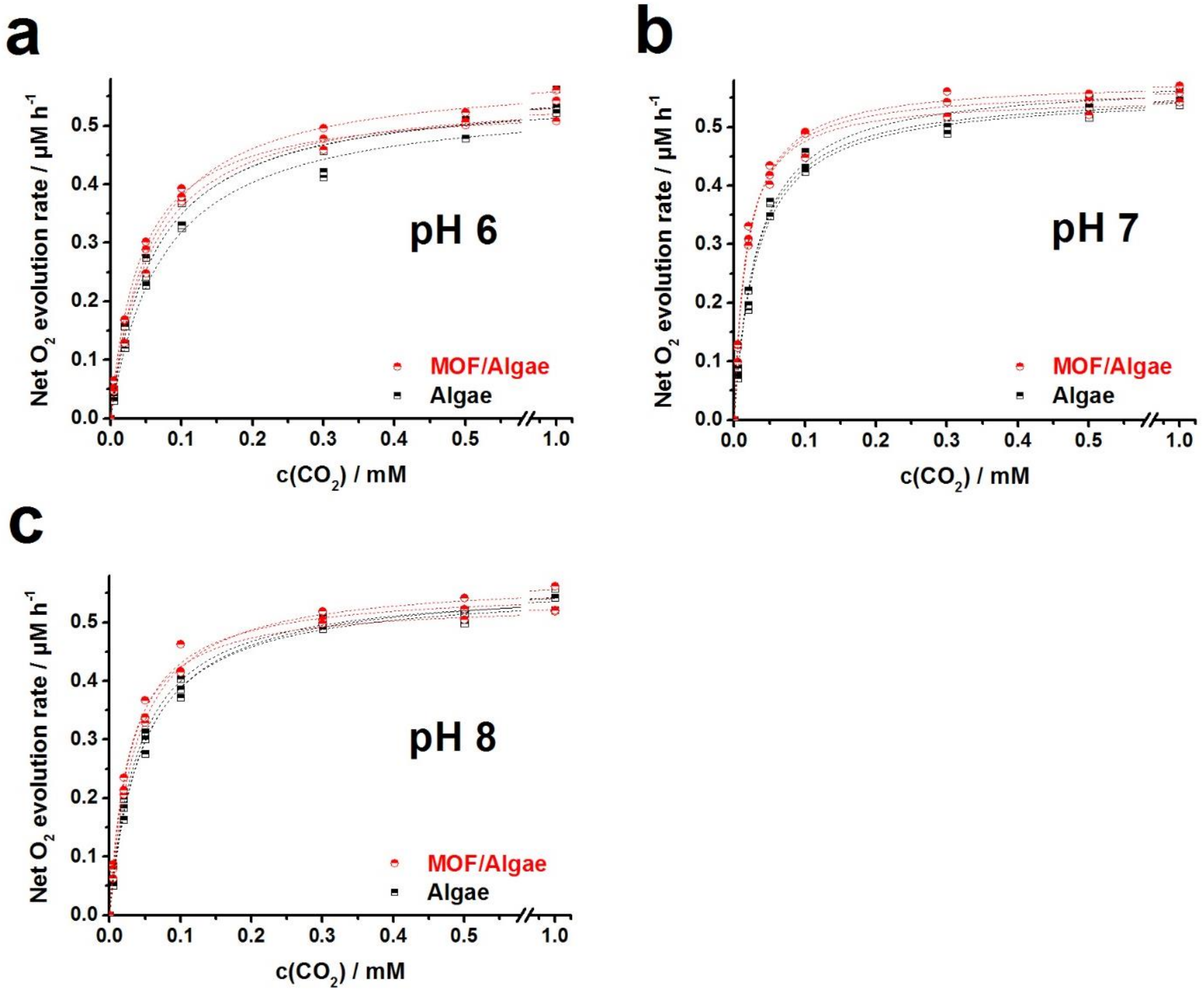

Supplementary Figure 22. pH effect on the biomass promotion of MOF assembled *C. pyrenoidosa*. The plot and the Michaelis-Menten fit of the net O_2 evolution rate versus the concentration of CO_2 for *C. pyrenoidosa* (Algae) and MOF/*C. pyrenoidosa* (MOF/Algae) cells grown at **(a)** pH 6, **(b)** pH 7 and **(c)** pH 8 under air (LC, 0.04%). Cell density of 1.0×10^7 cell mL^{-1} and the light source with a cutoff wavelength filter (under 50 μE m^{-2} s^{-1} irradiation, $\lambda > 600$ nm). Three independent experimental results are exhibited in each graph. Source data are provided as a Source Data file.

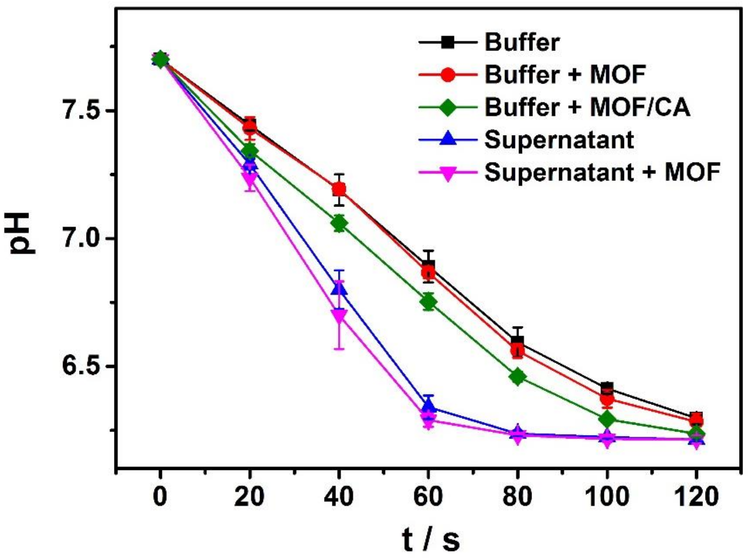

Supplementary Figure 23. Kinetic plots of the reaction that converts CO_2 to HCO_3^- in different environments. pH 7.0 20 mM HEPES buffer, the supernatant of microalgal suspension after two-day cultivation, and the addition of bare MOF or MOF pre-treated in supernatant, pH changes indicate the accumulation of products since proton generated simultaneously with HCO_3^- in this process. Cultivation conditions: temperature, 26°C; light intensity, 50 μE m^{-2} s^{-1}; 20 mL min^{-1} ambient air flow (LC); 24 hours continuous illumination; 20 mM HEPES buffer (initial pH 7.0). Error bars represent the standard deviation of the results from three biologically independent experiments. Source data are provided as a Source Data file.

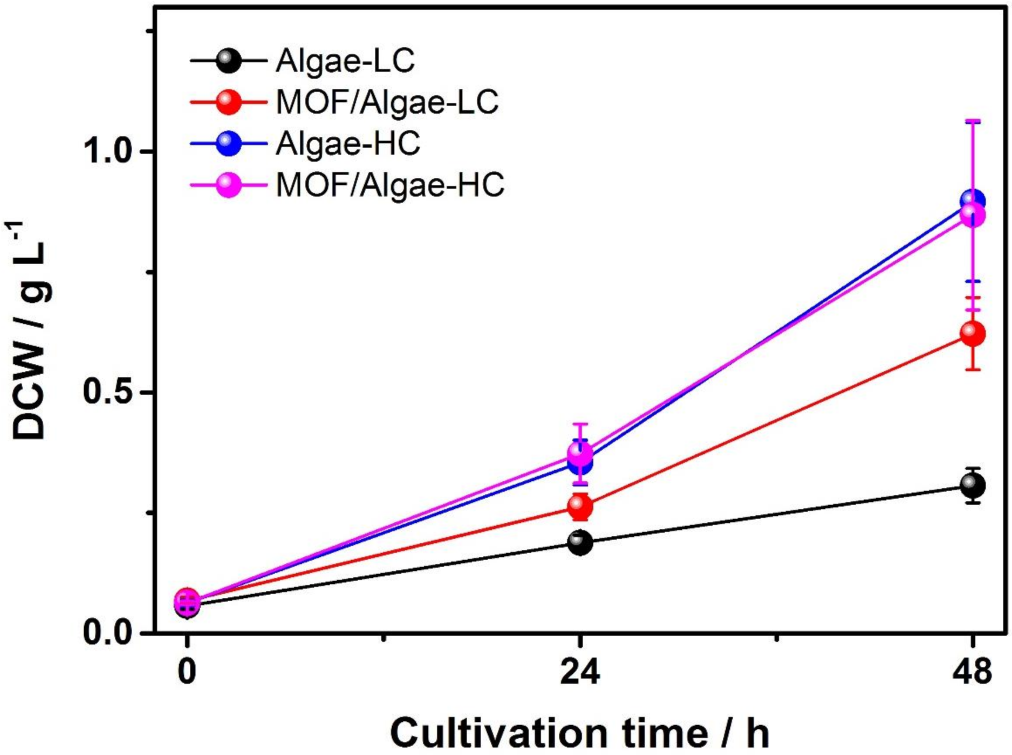

Supplementary Figure 24. Biomass growth curves of *C. pyrenoidosa* and MOF/*C. pyrenoidosa*. Cells were grown at pH 7 under air (LC, 0.04%) or 2% CO_2 (HC). Cultivation conditions: temperature, 26°C; light intensity, 50 μE m^{-2} s^{-1}; 20 mL min^{-1} gas flow; 24 hours continuous illumination. Error bars represent the standard deviation of the results from three biologically independent experiments. Source data are provided as a Source Data file.

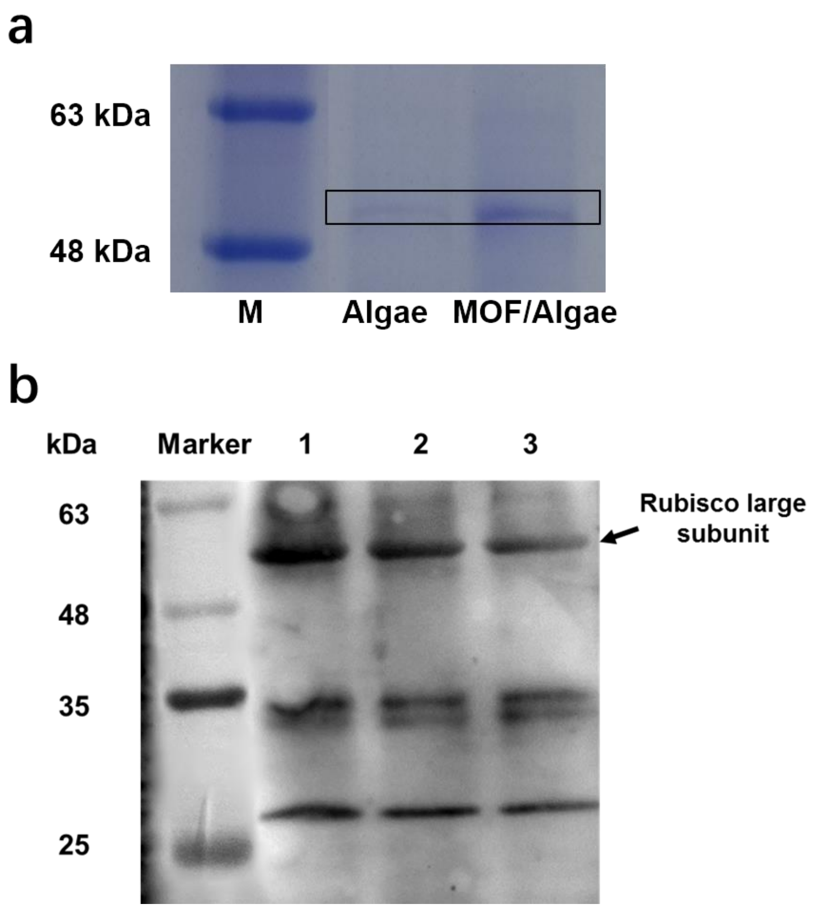

Supplementary Figure 25. Rubisco identification and quantification in *C. pyrenoidosa*. (a) The Coomassie brilliant blue stained SDS-PAGE analysis of Rubisco in bare *C. pyrenoidosa* and MOF/*C. pyrenoidosa* assembly. **(b)** Western blot for Rubisco in the homogenate of *C. pyrenoidosa*. The total protein amount, 1.73 μg; Cultivation conditions: temperature, 26°C; light intensity, 50 μE m^{-2} s^{-1}; 20 mL min^{-1} ambient air flow; 24 hours continuous illumination. Three numbers represent three biological replicas.

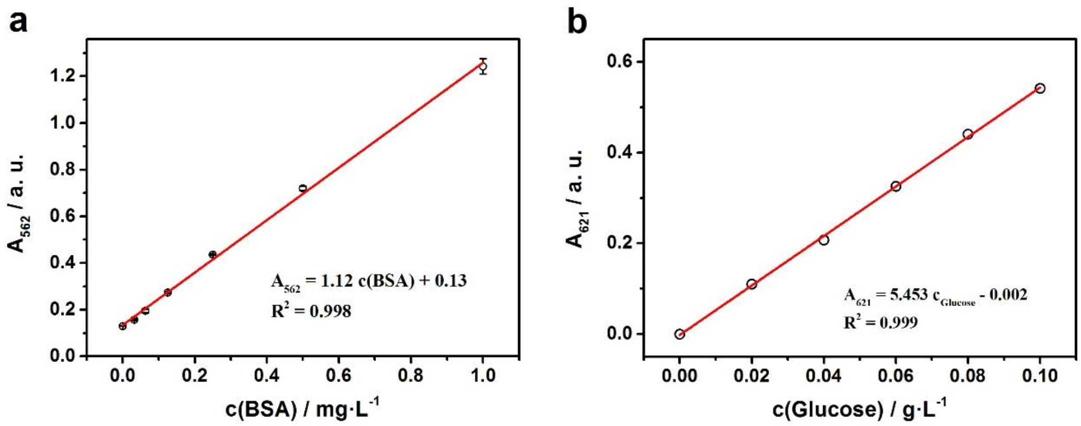

Supplementary Figure 26. Determination of protein and glucose contents in *C. pyrenoidosa*. The calibration curves of **(a)** protein that A_{562} versus the concentration of BSA standard solutions and **(b)** carbohydrate that A_{621} versus the concentration of glucose standard solutions. Source data are provided as a Source Data file.

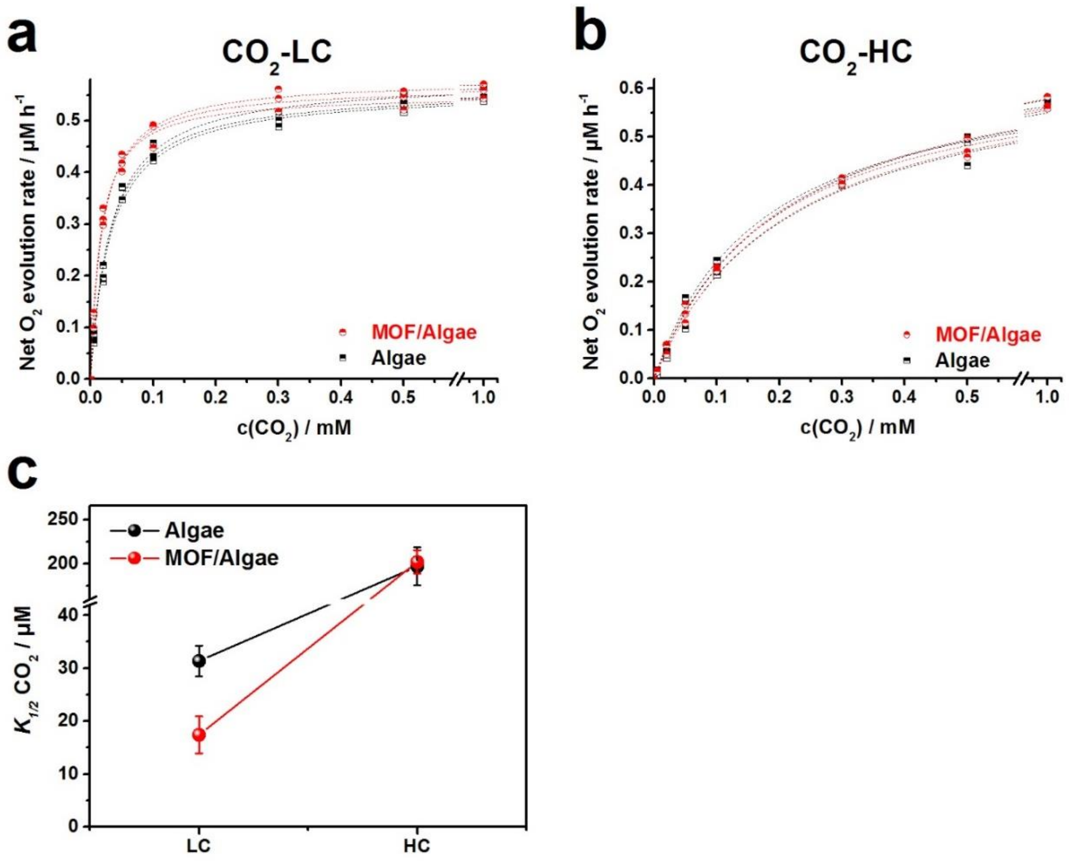

Supplementary Figure 27. CO_2 concentration effect on the biomass promotion of MOF assembled ***C. pyrenoidosa***. **(a-b)** The plot and the Michaelis-Menten fit of the net O_2 evolution rate versus the concentration of CO_2 for *C. pyrenoidosa* (Algae) and MOF/*C. pyrenoidosa* (MOF/Algae) cells grown under **(a)** air (LC, 0.04%) and **(b)** 2% CO_2 (HC) at pH 7. **(c)** The Michaelis constant $K_{1/2}$ values as calculated from the Michaelis-Menten fit of the plot of the net O_2 evolution rate versus the concentration of CO_2 for *C. pyrenoidosa* (Algae) and MOF/*C. pyrenoidosa* (MOF/Algae) cells grown at pH 7 under air (LC, 0.04%) or 2% CO_2 (HC). Cell density of 1.0×10^7 cell mL^{-1} and the light source with a cutoff wavelength filter (under 50 μE m^{-2} s^{-1} irradiation, $\lambda > 600$ nm). Error bars in **(c)** represent the standard deviation of $K_{1/2}$ values as fitted from three biologically independent experimental results. Source data are provided as a Source Data file.

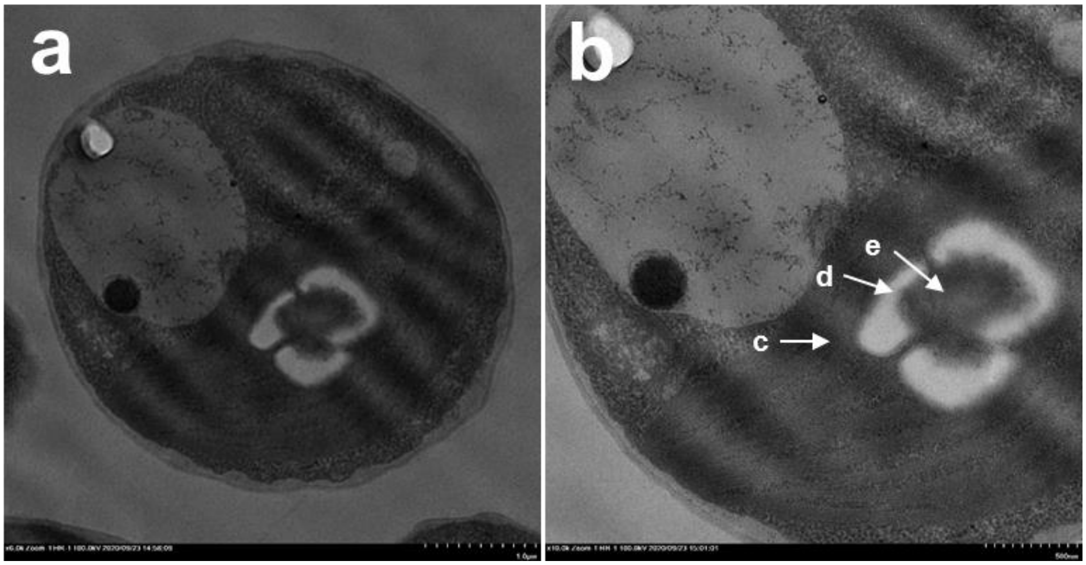

Supplementary Figure 28. Subcellular structures of *C. pyrenoidosa.* TEM images of the cross-section of *C. pyrenoidosa* at low (a) and high (b) magnifications. The arrows marked thylakoid membrane (c), starch sheath (d) and pyrenoid matrix (e) containing dense-packed Rubisco. The scale bars are 1 μm (a) and 500 nm (b), respectively.
