# Supplementary material for: Enhancing photosynthetic CO2 fixation by assembling metal-organic frameworks on Chlorella pyrenoidosa
Source: Nat Commun. 2023 Sep 2;14:5337. doi: 10.1038/s41467-023-40839-0 (PMC10475011; doi:10.1038/s41467-023-40839-0)
Supplement: Supplementary file 3 — Reporting Summary [file 41467_2023_40839_MOESM3_ESM.pdf]

nature portfolio

Corresponding author(s): Can Li

Last updated by author(s): Jul 28, 2023

Reporting Summary

Nature Portfolio wishes to improve the reproducibility of the work that we publish. This form provides structure for consistency and transparency in reporting. For further information on Nature Portfolio policies, see our Editorial Policies and the Editorial Policy Checklist.

Statistics

For all statistical analyses, confirm that the following items are present in the figure legend, table legend, main text, or Methods section.

n/a	Confirmed	
☐	☒	The exact sample size (*n*) for each experimental group/condition, given as a discrete number and unit of measurement
☐	☒	A statement on whether measurements were taken from distinct samples or whether the same sample was measured repeatedly
☐	☒	The statistical test(s) used AND whether they are one- or two-sided<br>*Only common tests should be described solely by name; describe more complex techniques in the Methods section.*
☒	☐	A description of all covariates tested
☒	☐	A description of any assumptions or corrections, such as tests of normality and adjustment for multiple comparisons
☐	☒	A full description of the statistical parameters including central tendency (e.g. means) or other basic estimates (e.g. regression coefficient) AND variation (e.g. standard deviation) or associated estimates of uncertainty (e.g. confidence intervals)
☒	☐	For null hypothesis testing, the test statistic (e.g. *F*, *t*, *r*) with confidence intervals, effect sizes, degrees of freedom and *P* value noted<br>*Give P values as exact values whenever suitable.*
☒	☐	For Bayesian analysis, information on the choice of priors and Markov chain Monte Carlo settings
☒	☐	For hierarchical and complex designs, identification of the appropriate level for tests and full reporting of outcomes
☒	☐	Estimates of effect sizes (e.g. Cohen's *d*, Pearson's *r*), indicating how they were calculated

Our web collection on statistics for biologists contains articles on many of the points above.

Software and code

Policy information about availability of computer code

Data collection	CO_2 adsorption isotherms of NH_2-MIL-101-Fe (298 K) were plotted by ASAP2020 physical adsorption analyzer (Micromeritics, USA). The adsorption of dissolved inorganic carbon (DIC) in MOF was determined by a TOC/TN Analyzer (Teledyne Tekmar, USA). Scanning electron microscopic characterizations were conducted on a JSM-7900F FESEM (JEOL, Japan). Chlorophyll fluorescence parameters were measured by Dual-PAM-100 (HeinzWalz).
Data analysis	Statistical analyses were conducted using OriginPro 9.

For manuscripts utilizing custom algorithms or software that are central to the research but not yet described in published literature, software must be made available to editors and reviewers. We strongly encourage code deposition in a community repository (e.g. GitHub). See the Nature Portfolio guidelines for submitting code & software for further information.

Data

Policy information about availability of data

All manuscripts must include a data availability statement. This statement should provide the following information, where applicable:

- Accession codes, unique identifiers, or web links for publicly available datasets
- A description of any restrictions on data availability
- For clinical datasets or third party data, please ensure that the statement adheres to our policy

The authors declare that the main data supporting the findings of this study are available within the article and its Supplementary Information files.

Human research participants

Policy information about studies involving human research participants and Sex and Gender in Research.

Reporting on sex and gender	Not applicable
Population characteristics	Not applicable
Recruitment	Not applicable
Ethics oversight	Not applicable

Note that full information on the approval of the study protocol must also be provided in the manuscript.

Field-specific reporting

Please select the one below that is the best fit for your research. If you are not sure, read the appropriate sections before making your selection.

- [x] Life sciences
- [ ] Behavioural & social sciences
- [ ] Ecological, evolutionary & environmental sciences

For a reference copy of the document with all sections, see nature.com/documents/nr-reporting-summary-flat.pdf

Life sciences study design

All studies must disclose on these points even when the disclosure is negative.

Sample size	All experiments were performed in three biologically independent experiments and samples.
Data exclusions	No data was excluded.
Replication	The experiments were repeated 3 times and the results are reproducible.
Randomization	The allocation is random, since all Chlorella pyrenoidosa cells have the same genetic background, and the sampling amount is large enough (10^7 cells per sampling).
Blinding	We are blinded to group allocation during data collection and analysis.

Reporting for specific materials, systems and methods

We require information from authors about some types of materials, experimental systems and methods used in many studies. Here, indicate whether each material, system or method listed is relevant to your study. If you are not sure if a list item applies to your research, read the appropriate section before selecting a response.

Materials & experimental systems

n/a	Involved in the study	
[ ]	[x]	Antibodies
[x]	[ ]	Eukaryotic cell lines
[x]	[ ]	Palaeontology and archaeology
[x]	[ ]	Animals and other organisms
[x]	[ ]	Clinical data
[x]	[ ]	Dual use research of concern

Methods

n/a	Involved in the study	
[x]	[ ]	ChIP-seq
[ ]	[x]	Flow cytometry
[x]	[ ]	MRI-based neuroimaging

Antibodies

Antibodies used	Rubisco Mouse Monoclonal Antibody；Horseradish-conjugated goat-anti-rabbit secondary' antibody
Validation	An anti-Rubisco monoclonal antibody (Catalog No. AG5359, Beyotime, China). https://www.beyotime.com/product/AG5359.htm； An horseradish-conjugated goat-anti-rabbit secondary antibody (Catalog No. A0208, Beyotime, China). https://www.beyotime.com/product/A0208.htm

Flow Cytometry

Plots

Confirm that:

- [x] The axis labels state the marker and fluorochrome used (e.g. CD4-FITC).
- [x] The axis scales are clearly visible. Include numbers along axes only for bottom left plot of group (a 'group' is an analysis of identical markers).
- [x] All plots are contour plots with outliers or pseudocolor plots.
- [x] A numerical value for number of cells or percentage (with statistics) is provided.

Methodology

Sample preparation	MOF, C. pyrenoidosa and MOF/C. pyrenoidosa were redispersed in BG-11 medium to be analyzed by flow cytometer.
Instrument	Sony SH800
Software	Cell Sorter Software version 2.1
Cell population abundance	The cell density of C. pyrenoidosa was 0.2 × 10^7 cells per milliliter.
Gating strategy	Rely on the preliminary FSC/SSC signal area.

- [x] Tick this box to confirm that a figure exemplifying the gating strategy is provided in the Supplementary Information.
